# The relationship between serum uric acid levels and glomerular ischemic lesions in patients with Immunoglobin A nephropathy-a analytical cross-sectional study

**DOI:** 10.1186/s12882-022-02880-x

**Published:** 2022-07-18

**Authors:** Bolong Fang, Yamin Yu, Xiaowei Dong, Lin Qi, Yan Wang, Fang Dai, Lan Wei, Yajie Kang

**Affiliations:** 1 Department of Nephrology, the 82Nd Group Military Hospital of the Chinese People’s Liberation Army, Baoding, 071000 Hebei China; 2 Department of Nephrology, Liaocheng People’s Hospital, No.67 Dongchang West Road, Shandong 25200 Liaocheng city, China

**Keywords:** IgA nephropathy, Hyperuricemia, Glomerular ischemic lesions

## Abstract

**Background:**

To investigate the relationship between serum uric acid levels and glomerular ischemic lesions in patients with immunoglobulin A nephropathy (IgAN) and the relevant risk factors.

**Methods:**

A total of 86 patients with IgAN and normal renal functions were divided into a hyperuricemia group and a normal serum uric acid group (control group). These patients were further divided into a glomerular ischemic lesions group and a non-glomerular ischemic lesions group (control group) based on the renal biopsy results. The relationship between serum uric acid levels and glomerular ischemic lesions was analysed.

**Results:**

In patients with IgAN, the prevalence or occurrence of glomerular ischemic lesions was significantly higher in the hyperuricemia group compared with the normal serum uric acid group. Elevated serum uric acid levels are independently associated with glomerular ischemic disease.

**Conclusion:**

Hyperuricemia in patients with IgAN may lead to glomerular ischemic lesions, and lowering serum uric acid levels may delay the progression of IgAN.

## Introduction

Immunoglobulin A nephropathy (IgAN) is the most common glomerular disease in the world [1], and it is also an important cause of end-stage renal disease. Hyperuricemia is an independent risk factor that affects the prognosis of IgAN [2]. It can lead to vascular endothelial dysfunction, but studies rarely report whether elevated serum uric acid levels aggravate renal ischemic injury in patients with IgAN and normal renal functions. In this paper, we analyse the relationship between the serum uric acid levels of patients with IgAN and glomerular ischemic lesions. Timely control and intervention against IgAN risk factors may decrease kidney damage and delay the progression of this disease. Previous studies suggest the crucial role of the complement system in the pathogenesis of IgAN [3]; therefore, quantification of complement factors in serum, urine or renal tissue can be a good marker for disease activity and prognosis.

## Methods

### Subjects and methods

#### General information

Patients with IgAN, normal renal function and average serum creatinine of (74.20 + 18.41) µmol/L, except those who had secondary IgANs such as Henoch-Schönlein purpura, systemic lupus erythematosus, human immunodeficiency virus infection and liver cirrhosis, were diagnosed using renal biopsy in our hospital between March 2016 and November 2020 and were enrolled in this study. There was a total of 86 patients: 41 males and 45 females between the ages of 16 and 67 years and with an average age of 37.98 ± 13.12 years.

## Methods

### Grouping

The patients were divided into a hyperuricemia group and a normal serum uric acid group based on serum uric acid levels. The diagnostic criteria for hyperuricemia were serum uric acid levels of > 420 µmol/L for males and > 357 µmol/L for females. The patients were further divided into a glomerular ischemic lesions group and a non-glomerular ischemic lesions group based on the renal biopsy results. The evaluation criteria for glomerular ischemic lesions were the presence of any glomerular ischemic sclerosis, ischemic atrophy or ischemic shrinkage based on renal biopsy. There were 40 patients in the glomerular ischemic lesions group: 28 males and 12 females between the ages of 23 and 66 years and with an average age of 41.53 ± 13.42 years. There were 46 patients in the non-glomerular ischemic lesions group: 13 males and 33 females between the ages of 16 and 67 years and with an average age of 34.89 ± 12.17 years.

### Detection indicators

serum uric acid levels, body weight, blood pressure, 24-h proteinuria (PRO) levels, blood cholesterol levels, triglycerides, high-density lipoprotein (HDL) and low-density lipoprotein (LDL) in the two groups.

### Statistical analysis

The SPSS 22.0 software was used for statistical analysis. Count data was tested with the χ^2^ test, and measurement data was tested with the Student’s t-test and the results are expressed as the mean ± standard deviation (x ± s). To determine the independent risk factors for glomerular ischemic injury, binary logistic regression analysis was used. A two-tailed *P* < 0.05 was considered statistically significant.

## Results

The relationship between IgAN serum uric acid levels and glomerular ischemic lesions.

The prevalence or occurrence of glomerular ischemic lesions in the IgAN with hyperuricemia group (68.0%) was significantly higher than in the normouricemia group (37.7%) (χ^2^ = 6.542, *P* < 0.05, Table 1).

**Table 1:** Relationship between serum uric acid level and glomerular ischemic lesions in IgAN patients

Group	Number of cases	Male/female	Age	Serum uric acid water Level (μmol/L)	Number of ischemic lesions	Incidence of ischemic lesions	Heart rate ( beats/min)	Glycaemia ( mg/dL)	Cholesterol ( mg/dL)
Hyperuricemia group	25	14/11	62	454.56 ± 70.60	17	68.0%*	80.08 ± 12.31	175.94 ± 65.57	191.48 ± 53.21
Normouricemia group	61	27/34	61.39	304.97 ± 60.50	23	37.7%	78.88 ± 11.48	181.8 ± 81.49	189.01 ± 55.18

*Note*: Compared with the normouricemia group, *p < 0.05

When serum uric acid levels in patients with IgAN increased above 360 µmol/L, the prevalence or occurrence of glomerular ischemic lesions significantly increased. Recently, some studies reports hyperuricemia is induced by alterations in purine metabolism and increased serum uric acid is associated with increased risk of developing hyperuricemia, which is consistent with the conclusion shown by the article. Moreover, glomerular ischemic lesions were associated with serum uric acid levels (χ^2^ = 20.302, *P* < 0.05, *r* = 0.437, Table 2).

**Table 2:** Relationship between serum uric acid level and glomerular ischemic lesions in IgAN patients

Group	Number of cases	Male/female	Age	Serum uric acid level	Number of ischemic lesions	Prevalence of ischemic lesions
≤ 360	48	14/34	36.21 ± 12.27	282.60 ± 46.70	12	25.0%^#^
360–420 ≥ 420	21 17	8/13 3/14	39.76 ± 13.98 40.76 ± 14.36	384.71 ± 16.03 489.59 ± 58.10	15 13	71.4%* 76.5%*

*Note*: *P < 0.05 compared with the prevalence of ischemic lesions in the group with serum uric acid ≤ 360 µmol/L; #*P* < 0.05 compared with the prevalence of ischemic lesions in the group with serum uric acid 360–420 µmol/L

Comparison of clinical indicators between the IgAN with glomerular ischemic lesions group and the IgAN without glomerular ischemic lesions group: Uric acid levels, body weight, systolic blood pressure and diastolic blood pressure in the glomerular ischemic lesions group were significantly higher than those in the control group (*P* < 0.05). HDL in the glomerular ischemic lesions group was significantly lower than in the control group (*P* < 0.05) (Table 3).

**Table 3:** Comparison of uric acid, body weight, blood lipids, Proteinuria, and blood pressure between the glomerular ischemic lesion group and the non-glomerular ischemic lesion group

Item	Glomerular ischemic lesion group	Non-glomerular ischemic lesion group	*P* value
Number of cases (%)	40 (46.51%)	46 (53.49%)	86 (100%)
Uric acid (μmol/L)	400.9 ± 85.29	302.8 ± 74.03	0.000
Body weight (kg)	74.50 ± 12.70	62.60 ± 10.37	0.000
Gender (male / female)	28/12	13/33	0.000
Age (years)	41.53 ± 13.42	34.89 ± 12.17	0.018
Cholesterol (mmol/L)	5.42 ± 1.21	5.90 ± 2.71	0.317
Triglycerides (mmol/L)	1.99 ± 1.11	1.78 ± 1.05	0.397
HDL (mmol/L)	1.17 ± 0.34	1.40 ± 0.48	0.020
LDL (mmol/L)	3.43 ± 1.10	3.72 ± 2.24	0.473
Proteinuria (g/24 h)	1.30 ± 1.17	2.00 ± 2.36	0.095
Systolic blood pressure (mmHg)	132.44 ± 19.38	120.76 ± 14.53	0.002
Diastolic blood pressure (mmHg)	86.18 ± 14.48	76.70 ± 10.63	0.003

Multivariate analysis of glomerular ischemic lesions: Based on the presence or absence of glomerular ischemic lesions in the 86 patients with IgAN, logistic regression was performed to analyse the influence of the factors that had *P* < 0.05 in univariate analysis. The results showed that serum uric acid levels were an independent risk factor for glomerular ischemic lesions (Table 4).

**Table 4:** Factors correlated with glomerular ischemic lesions

Variable	OR (95% CI)	*P* value
Uric acid	1.010(1.000,1.019)	0.042
Body weight	1.035(0.980,1.094)	0.214
Gender	0.431(0.107,1.738)	0.237
Age	1.043(0.991,1.098)	0.111
HDL-cholesterol	0.504(0.095, 2.661)	0.419
Systolic blood pressure Diastolic blood pressure	1.018(0.959, 1.081) 0.977(0.897, 1.064)	0.560 0.593

## Discussion

Because renal insufficiency can affect the excretion of serum uric acid, resulting in hyperuricemia, and severe renal pathological changes may interfere with the diagnosis of renal ischemic injury, we studied patients with IgAN and normal renal function. The results suggested that the prevalence or occurrence of glomerular ischemic lesions in patients with IgAN and hyperuricemia was significantly higher than in the normouricemia group. As serum uric acid levels increased, the prevalence or occurrence of glomerular ischemic lesions increased, and serum uric acid levels were an independent risk factor for glomerular ischemic lesions. In patients with IgAN, hyperuricemia may be associated with glomerular ischemia and participate in the process of renal injury.

There is evidence that persistent hyperuricemia can cause renal tissue changes, such as arteriolonephrosclerosis, glomerulosclerosis and renal tubular lesions, leading to chronic kidney disease [4, 5]. As Russo E et al. reported in a retrospective study, Serum uric levels are independently associated with AD and poor prognosis in patients with IgAN [6].One of the mechanisms serum uric acid aggravates renal ischemic injury may be the activation of the renin–angiotensin system. Renal artery stenosis is an important mediator of renal disease progression. It affects renal hemodynamics, increases glomerular perfusion pressure and promotes renal vascular smooth muscle hyperplasia, endothelial cell fibrosis and inflammatory cell infiltration [7, 8]. Uric acid can activate extracellular signal–regulated kinase (ERK1/2), accompanied by de novo induction of cyclooxygenase 2 (COX2) and local thromboxane synthesis. It can upregulate the mRNA levels of platelet-derived growth factor (PDGF) A and C chains and platelet-derived growth factor (PDGF)-α receptor. Uric acid can also stimulate monocytes to synthesise monocyte chemoattractant protein 1, which is a key chemotactic factor for vascular diseases and atherosclerosis. These inflammatory reactions may cause vascular smooth muscle damage and proliferation [9, 10]. Animal experiments have shown that hyperuricemia can directly promote vascular smooth muscle hyperplasia and thicken the afferent arterioles, and it can cause arterial contraction when serum uric acid levels are slightly elevated [11]. Other animal experiments show that In the kidney of hyperuricemic rats, endothelial staining in peritubular capillaries (PTC) was substantially decreased with de-novo expression of α-smooth muscle actin in endothelial cells of PTC. Serum uric induced a phenotypic transition of epithelial and endothelial cells via an induction of oxidative stress and glycocalyx shedding, which could be one of the mechanisms of uric acid-induced kidney disease [12]. Uric acid can damage the ability of endothelial cells to produce nitric oxide (NO), weakening vasodilation [13], enhancing endothelial cell oxidativ e stress and promoting endothelial cell apoptosis [14]. Changes in these blood vessels may cause stenosis or occlusion of small arteries, leading to glomerular ischemic pathological changes and further aggravating kidney injury. As Dong et al. reported in a study, Arterial-arteriolar sclerosis (AS) in patients with IgAN was independently associated with the poor prognosis, In the subgroup analysis, patients presenting with AS and higher uric acid had a significant trend for a shorter time to reach the end point [15].The narrowing of the lumen of small blood vessels can further enhance renin activity, resulting in a vicious cycle.

The incidence of hyperuricemia in the glomerular ischemic lesions group was significantly higher than in the non-glomerular ischemic lesions group. This may be because renal ischemia can lead to hypoxia in local tissues, increasing the production of lactic acid. Excessive lactic acid excretion competitively inhibits uric acid excretion, resulting in uric acid retention in the body and reducing urate clearance through the action of lactic acid [16]. Low renal blood flow perfusion stimulates the reabsorption of uric acid. Ischemia can also lead to increased uric acid synthesis; in an ischemic environment, ATP is decomposed into adenine and xanthine and more xanthine oxidase is generated.

This study has certain limitations. We only measured serum uric acid once; therefore, the assessment of the relationship between serum uric acid levels and IgAN glomerular ischemic lesions was not as accurate as possible. Furthermore, beside renal factors, excessive alcohol intake, a high-purine diet and the application of diuretics can also lead to hyperuricemia, and this study did not consider these factors. Finally, when serum uric acid concentration exceeds 410 µmol/L, uric acid in the plasma is saturated (at pH 7.4, temperature 37 °C and serum sodium under normal conditions). If serum uric acid concentration reaches saturation, these substances are prone to form crystals and accumulate in soft tissues. Therefore, some male patients may have hyperuricemia even if serum uric acid concentration is lower than 420 µmol/L.

In summary, serum uric acid levels of patients with IgAN are closely correlated with glomerular ischemic lesions, and the two may affect each other. Reducing the serum uric acid level may reduce the degree of glomerular ischemic injury.

## Data Availability

All data generated or analyzed during this study are included in this published article.
