# Correction: The relationship between serum uric acid levels and glomerular ischemic lesions in patients with Immunoglobin A nephropathy-a analytical cross-sectional study

**DOI:** 10.1186/s12882-023-03425-6

**Published:** 2023-12-12

**Authors:** Bolong Fang, Yamin Yu, Xiaowei Dong, Lin Qi, Yan Wang, Fang Dai, Lan Wei, Yajie Kang

**Affiliations:** 1 Department of Nephrology, the 82Nd Group Military Hospital of the Chinese People?s Liberation Army, Baoding, 071000 Hebei China; 2 Department of Nephrology, Liaocheng People?s Hospital, No.67 Dongchang West Road, Shandong 25200 Liaocheng city, China

**Correction: BMC Nephrology (2022) 23:255**

10.1186/s12882-022-02880-x

Following publication of the original article [1], the authors informed us that would like to update the Funding section. The correct Funding is given below:

## References

[1] Fang, et al. BMC Nephrol. 2022;23:255. 10.1186/s12882-022-02880-x 10.1186/s12882-022-02880-x PMC9295508 35850659

